# Multinational evaluation of the BioFire® FilmArray® Pneumonia *plus* Panel as compared to standard of care testing

**DOI:** 10.1007/s10096-021-04195-5

**Published:** 2021-03-02

**Authors:** Christine C. Ginocchio, Carolina Garcia-Mondragon, Barbara Mauerhofer, Cory Rindlisbacher, and the EME Evaluation Program Collaborative, L. Forcelledo, J. Fernández, R. Lienhard, H. Kerschner, G. M. Rossolini, L. Armand-Lefèvre, C. d’Humières, E. Cambau, H. Benmansour, R. Cavallo, M. Altwegg, L. Berlinger, R. Bonnet, P. Saint-Sardos, C. Meex, J. P. Lavigne, N. Leveque, L. Broutin, V. Cattoir, G. Auger, H. Pereira, Y. Paitan, A. Verroken, H. Pailhoriès, C. Lemarié, G. Martinetti-Lucchini, S. Frigerio Malossa, M. Sanguinetti, T. Spanu, F. Vandenesch, C. Poyart, J. Loubinoux, J. P. Mira, S. Bonacorsi, A. Cointe, P. Muñoz, M. Kestler, C. Esteva, X. Queralt, J. García-Rodríguez, M. D. Gómez, J. L. López-Hontangas, V. Ghisetti, E. Burdino, S. Schubert, A. Mencacci, F. Allegrucci, W. Rozemeijer, N. Paternotte, A. Allard, M. C. Re, S. Ambretti, M. Skov, C. N. Agergaard, P. Subudhi, T. A. Wichelhaus, A. Egli, V. Hinic, A. Alcock, K. Banavathi, C. Tiberio, G. Ruocco, L. Atripaldi

**Affiliations:** 1 BioFire Diagnostics, LLC, 515 Colorow Way, Salt Lake City, UT 84108 USA; 2 bioMérieux, Salt Lake City, UT USA; 3 bioMérieux, Marcy l’Etoile, France

**Keywords:** Pneumonia, CAP, HAP, VAP, Diagnosis, BioFire Pneumonia *plus* Panel

## Abstract

This study compared standard of care testing (SOC) to BioFire® FilmArray® Pneumonia *plus* Panel (PN*plus*). PN*plus* detects 15 bacteria with semiquantitative log bin values, 7 antibiotic resistance markers, three atypical bacteria (AB), and eight viral classes directly from bronchoalveolar lavage-like specimens (BLS) and sputum-like specimens (SLS). Fifty-two laboratories from 13 European countries and Israel tested 1234 BLS and 1242 SLS with PN*plus* and SOC. Detection rates and number of pathogens/samples were compared for PN*plus* pathogens. PN*plus* bin values and SOC quantities were compared. Three thousand two hundred sixty-two bacteria in PN*plus* were detected by PN*plus* and/or SOC. SOC detected 57.1% compared to 95.8% for PN*plus* (*p* ≤ 0.0001). PN*plus* semiquantitative bin values were less than SOC, equal to SOC, or greater than SOC in 5.1%, 25.4%, and 69.6% of results, respectively. PN*plus* bin values were on average ≥ 1 log than SOC values (58.5% 1–2 logs; 11.0% 3–4 logs). PN*plus* identified 98.2% of MRSA and SOC 55.6%. SOC detected 73/103 AB (70.9%) and 134/631 viruses (21.2%). PN*plus* detected 93/103 AB (90.3%) and 618/631 viruses (97.9%) (*p* ≤ 0.0001). PN*plus* and SOC mean number of pathogens/samples were 1.99 and 1.44, respectively. All gram-negative resistance markers were detected. PN*plus* and SOC results were fully or partially concordant for 49.1% and 26.4% of specimens, respectively. PN*plus* was highly sensitive and detected more potential pneumonia pathogens than SOC. Semiquantification may assist in understanding pathogen significance. As PN*plus* generates results in approximately 1 h, PN*plus* has potential to direct antimicrobial therapy in near real time and improve antimicrobial stewardship and patient outcomes.

**Supplementary Information:**

The online version contains supplementary material available at 10.1007/s10096-021-04195-5.

## Introduction

Determining the etiology of community-acquired pneumonia (CAP), hospital-associated pneumonia (HAP), and ventilator-associated pneumonia (VAP) can be complicated [1]. Based on traditional test methods such as Gram stain and culture, diagnostic yield can be low [2–4]. Additionally, poor specimen quality may yield inconclusive or difficult to interpret results, sampling may require an invasive procedure, and/or patients may be on empiric therapy prior to specimen collection, reducing diagnostic yield [5]. Although international guidelines from the Infectious Diseases Society of America (IDSA)/American Thoracic Society (ATS) and the European Respiratory Society (ERS) recommend diagnostic testing for moderate to severe CAP [6, 7], actual compliance with guidelines can be low (16.7% and 23.9%, respectively) and differs by geography and disease severity [8]. Aside from influenza A/B, often no viral testing is performed, despite evidence that other viruses are a significant cause of CAP in all age patients [9–11]. Consequently, broad use of empiric antibiotic treatment for undiagnosed viral infections has been associated with increased antibiotic resistance globally [12–14].

In HAP and VAP, empiric therapy often includes broad-spectrum antibiotics for both gram-positive and gram-negative bacteria due to the risk of infection with multidrug-resistant pathogens [15, 16]. Identification of specific pathogen(s) is of increasing importance to allow prompt initiation of targeted and effective therapy and should have a positive impact on antimicrobial resistance and consequently on healthcare expenditure, while reducing risks for adverse events such as renal impairment and development of *Clostridium difficile* disease [17–19].

To address these unmet diagnostic needs and to provide a solution for prompt initiation of targeted therapy, the BioFire® FilmArray® Pneumonia (PN)/Pneumonia *plus* (PN*plus*) Panels (BioFire Diagnostics, LLC, Salt Lake City, UT) were developed. BioFire PN/PN*plus* Panels are US Food and Drug Administration (FDA) cleared and CE-IVD marked highly multiplexed nested polymerase chain reaction tests that identify the common causes of CAP, HAP, and VAP [20]. BioFire PN/PN*plus* Panels contain identical test reagents; however, results for Middle East respiratory syndrome coronavirus (MERS-CoV) are masked by the software for BioFire PN Panel. BioFire PN*/*PN*plus* Panels detect 15 bacteria with a semiquantitative log value (10^4^, 10^5^, 10^6^, ≥ 10^7^), three atypical bacteria (*Legionella pneumophila* [all serotypes], *Mycoplasma pneumoniae*, *Chlamydia pneumoniae*), viruses reported as eight groups (adenovirus, coronaviruses [OC43, NL63, HKU-1, 229E], MERS-CoV [BioFire PN*plus* Panel], human metapneumovirus, human rhinovirus/enterovirus [HRV/EV], influenza A, influenza B, parainfluenza viruses, respiratory syncytial virus), and 7 genetic markers of antibiotic resistance (*mecA/C* and MREJ, *bla*_CTX-M_, *bla*_KPC_, *bla*_VIM_, *bla*_OXA-48-like_, *bla*_IMP_, *bla*_NDM_). BioFire PN/PN*plus* Panels are intended for use in persons of all ages and in various settings, including outpatient, emergency department, and hospitalized. The tests are validated for lower respiratory tract specimens, including sputum-like specimens (SLS) (induced, expectorated sputa, endotracheal aspirates [ETA]) and bronchoalveolar lavage (BAL)-like specimens (BLS) (BAL, mini-BAL). Specimen preprocessing is not required, test setup is approximately 5–10 min, and time to results is approximately 1 h.

This multicenter study assessed BioFire PN*plus* Panel performance as compared to standard of care testing (SOC) performed at a variety of institutions with variable test utilization and laboratory practices.

## Materials and methods

### Clinical sites, specimens, and SOC testing

Forty-eight academic medical center laboratories and four independent medical laboratories from 13 countries (Austria, Belgium, Denmark, Israel, Italy, France, Germany, Netherlands, Portugal, Spain, Sweden, Switzerland, and the UK) tested 2476 unique specimens (1234 BLS; 1242 SLS) from adult and pediatric patients suspected of pneumonia (Supplemental Table 7). Specimen selection was at discretion of study site. SOC was performed per institutional policies and healthcare provider prescription. SOC varied by site and all included bacterial culture and phenotypic susceptibility testing when indicated. Additional test methods were at the discretion of the laboratory and may have included additional cultures (fungal, viral, mycobacterial cultures) as needed, urinary antigen testing (*Streptococcus pneumoniae*, *L. pneumophila*), immunofluorescence tests, and nucleic acid amplification tests (NAATs) for selected bacteria, viruses, and antibiotic resistance markers. Methicillin-susceptible *Staphylococcus aureus* (MSSA) was differentiated from methicillin-resistant *S. aureus* (MRSA) using phenotypic methods, *mec*A NAAT, and/or PBP2a latex agglutination at the discretion of the laboratory. The investigators were instructed to perform the BioFire PN*plus* Panel in accordance with the manufacturer’s instruction for use. Specimens were split and BioFire PN*plus* Panel testing was performed using either fresh specimens or from a frozen aliquot. Data was deidentified, no protected health information was provided, and participation was in accordance with local institutional ethical guidelines.

BioFire PN*plus* and SOC results were compared for the following:

1. Detection of BioFire PN*plus* Panel pathogens by BioFire PN*plus* Panel and SOC: Results were evaluated for all specimens and by specimen types. SOC detected or not detected was determined in consideration of all test results reported. SOC *C. pneumoniae*, *L. pneumophila*, *M. pneumoniae*, and viral results were counted as not detected when a negative or no result was provided as patterns of testing varied extensively by institution. All SOC results were considered true positive. Results of BioFire PN*plus* Panel were considered true positive or true negative based on performance data established in US FDA clinical studies [20]. Mean numbers of BioFire PN*plus* Panel pathogens per specimen, rate, and composition of coinfections were compared to SOC. No discordant analyses were performed due to large numbers of isolates that would have been referred to a supplemental PCR. Additionally, most laboratories would not have another culture-independent method to confirm or refute results and the study was designed to mimic real-life interpretation of data.
2. Number and type of microorganisms reported by SOC not on BioFire PN*plus* Panel: Organisms considered always as normal flora (ex viridans streptococci) were excluded from analysis, and remaining organisms were classified as potential pathogens and questionable pathogens.
3. BioFire PN*plus* Panel and SOC results were evaluated for overall concordance. For common bacteria identified by routine culture, positive percent agreement (PPA) and negative percent agreement (NPA) for on-panel pathogens were calculated. The negative predictive value (NPV) for BioFire PN*plus* on panel pathogens and off panel pathogens was calculated.
4. Comparison of semiquantitative BioFire PN*plus* Panel and SOC values for bacteria: SOC reporting varied, including no quantification, semiquantitative descriptive (rare, few, moderate, many, etc.), semiquantitative numerical (1+, 2+, 3+, 4+), and quantitative culture in log values (10^2^–10^8^ CFU/mL). To equate SOC to BioFire PN*plus* Panel bin values, standardized reference values (SRV) were established as defined in Supplemental Table 8. Data was evaluated for all SOC and BioFire PN*plus* Panel comparisons (*n* = 1297) and for a subset of comparisons where quantitative numerical culture values were provided (*n* = 903).

### Statistical analysis

*p* values were calculated using a 2-specimen test for equality of proportions with a continuity correction. BioFire PN*plus* Panel PPA, NPA, and NPV for the common bacterial pathogens were calculated using the Clopper-Pearson method.

## Results

### Detection of BioFire PN*plus* Panel pathogens by BioFire PN*plus* and SOC

**Fig. 1:**
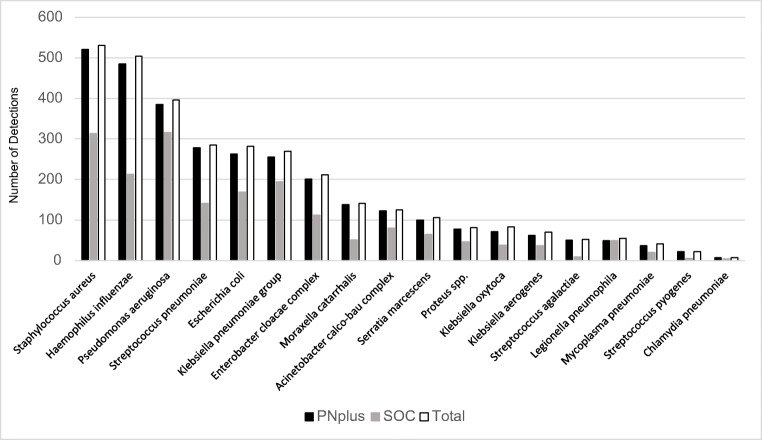
Comparison of composite total, BioFire Pneumonia *plus* Panel (PN*plus*) and standard of care (SOC) bacterial detections for all specimen types

Of the 2476 specimens tested with BioFire PN*plus*, 13 specimens (0.53%) gave invalid results (5 SLS; 8 BLS) leaving 2463 specimens (1237 SLS, 1226 BLS) evaluable. BioFire PN*plus* Panel detected one or more pathogens in 1875 specimens (76.13%) and SOC in 1380 specimens (56.03%) (*p* ≤ 0.0001) (Fig. 1). In total, 3893 bacteria and viruses were detected by at least one method (Table 1). BioFire PN*plus* Panel detected 3743/3893 pathogens (96.15%) compared to 1995/3893 pathogens (51.25%) for SOC (*p* ≤ 0.0001). BioFire PN*plus* Panel and SOC concordance for all pathogens detected was 47.39%. Sensitivities of BioFire PN*plus* Panel detections ranged from 85.54 to 100%, and sensitivities of SOC detections ranged from 8.89 to 100%.

**Table 1:** Summary of total, BioFire Pneumonia *plus* (PN*plus*) Panel, and standard of care (SOC) detections for all pathogens and for all specimen types

	Number SOC (+) PN*plus* (+)	Number SOC (−) PN*plus* (+)	Number SOC (+) PN*plus* (−)	Number total (+)	Number PN*plu*s (+)	Number SOC (+)	Percentage (%) detected PN*plus*	Percentage (%) detected SOC	*p* value^a^
*Acinetobacter calcoaceticus-baumannii* complex	78	45	2	125	123	80	98.40	64.00	< 0.0001*
Adenovirus	3	41	1	45	44	4	97.78	8.89	< 0.0001*
*Chlamydia pneumoniae*	4	3	0	7	7	4	100.00	57.14	0.19
Coronaviruses^b^	5	46	1	52	51	6	98.08	11.54	< 0.0001*
*Enterobacter cloacae* complex	102	99	10	211	201	112	95.26	53.08	< 0.0001*
*Escherichia coli*	150	113	19	282	263	169	93.26	59.93	< 0.0001*
*Haemophilus influenzae*	194	291	19	504	485	213	96.23	42.26	< 0.0001*
Human metapneumovirus	5	5	1	11	10	6	90.91	54.55	0.15
Rhinovirus/enterovirus	57	308	3	368	365	60	99.18	16.30	< 0.0001*
Influenza A	24	20	1	45	44	25	97.78	55.56	< 0.0001*
Influenza B	2	0	0	2	2	2	100	100	1.00
*Klebsiella aerogenes*	29	33	8	70	62	37	88.57	52.86	< 0.0001*
*Klebsiella oxytoca*	26	45	12	83	71	38	85.54	45.78	< 0.0001*
*Klebsiella pneumoniae* group	179	76	15	270	255	194	94.44	71.85	< 0.0001*
*Legionella pneumophila*	43	6	6	55	49	49	89.09	89.09	1.00
*Moraxella catarrhalis*	48	90	3	141	138	51	97.87	36.17	< 0.0001*
*Mycoplasma pneumoniae*	16	21	4	41	37	20	90.24	48.78	0.0001*
Parainfluenza viruses^c^	14	49	5	68	63	19	92.65	27.94	< 0.0001*
*Proteus* spp.	43	35	3	81	78	46	96.30	56.79	< 0.0001*
*Pseudomonas aeruginosa*	305	80	11	396	385	316	97.22	79.80	< 0.0001*
Respiratory syncytial virus	11	28	1	40	39	12	97.50	30.00	< 0.0001*
*Serratia marcescens*	58	42	6	106	100	64	94.34	60.38	< 0.0001*
*Staphylococcus aureus*	303	218	10	531	521	313	98.12	58.95	< 0.0001*
*Streptococcus agalactiae*	7	43	2	52	50	9	96.15	17.31	< 0.0001*
*Streptococcus pneumoniae*	134	144	7	285	278	141	97.54	49.47	< 0.0001
*Streptococcus pyogenes*	5	17	0	22	22	5	100	22.73	< 0.0001
Total	1845	1898	150	3893	3743	1995	96.15	51.25	< 0.0001

Abbreviations: (+): positive; (−): negative

^a^Significant *p* value* ≤ 0.05

^b^Coronaviruses: total includes OC43, NL63, 229E, and HKU-1

^c^Parainfluenza viruses: total includes types 1, 2, 3, and 4

For all specimen types, 3262 bacteria were detected by at least one method (Table 1). BioFire PN*plus* Panel detected 3125/3262 bacteria (95.77%) and SOC 1861/3262 (57.05%) (*p* ≤ 0.0001). BioFire PN*plus* Panel and SOC concordance was 52.85%. The most frequently detected bacteria per sample by BioFire PN*plus* Panel were *S. aureus* (21.15%), *Haemophilus influenzae* (19.69%), and *Pseudomonas aeruginosa* (15.63%) and by SOC were *P. aeruginosa* (12.83%), *S. aureus* (12.71%), and *H. influenzae* (8.65%).

**Fig. 2:**
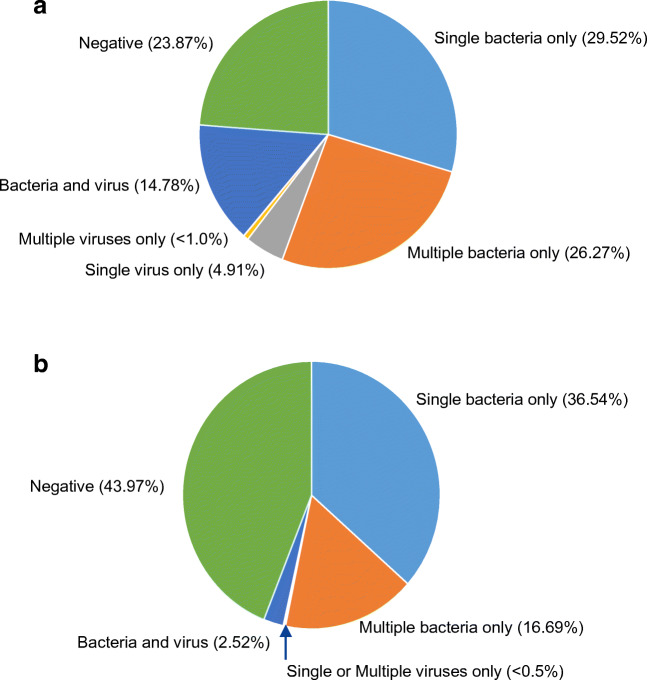
**a** Distribution of pathogens detected by BioFire Pneumonia *plus* Panel (PN*plus*) for all specimen types. **b** Distribution of pathogens detected by standard of care (SOC) for all specimen types

For BLS, 1358 bacteria were detected by at least one method (Supplemental Table 9). BioFire PN*plus* Panel identified 1288/1358 bacteria (94.85%) compared to 841/1358 (61.93%) for SOC (*p* ≤ 0.0001). BioFire PN*plus* Panel and SOC concordance was 56.77%. For SLS, 1904 bacteria were detected by at least one method (Supplemental Table 10). BioFire PN*plus* identified 1836/1904 (96.43%) bacteria and SOC 1020/1904 (53.57%) (*p* ≤ 0.0001). BioFire PN*plus* Panel and SOC concordance was 50%. PPAs for the individual PN*plus* common bacterial pathogens ranged from 68.42 to100% (mean 92.90%; 95% confidence interval [CI] 91.61 to 94.04%) for all specimens combined; 50.0 to 100% (mean 91.77%; 95% CI 89.66 to 93.56%) for BLS; and 80.95 to 100% (mean 93.74%; 95% CI 92.03 to 95.18% ) for SLS.

NPAs for the individual BioFire PN*plus* Panel common bacterial pathogens ranged from 87.07 to 99.31% (mean 96.10%; 95% CI 95.89 to 96.30%) for all specimens combined; 90.18 to 99.43% (mean 97.14%; 95% CI 96.89 to 97.39%) for BLS; and 83.86 to 99.19% (mean 94.98%; 95% CI 94.73 to 95.37%) for SLS. NPVs for the individual BioFire PN*plus* Panel common bacterial pathogens ranged from 99.04 to 99.96% (mean 99.63%; 95% CI 99.56 to 99.68%) for all specimens combined; 99.0 to 99.92% (mean 99.61%; 95% CI 99.51 to 99.69%) for BLS; and 99.15 to 99.94% (mean 99.64%; 95% CI 99.54 to 99.71%) for SLS. This evaluation did not consider the quantity of the common bacterial pathogens reported and the NPA and NPV may in practice be higher based on laboratory sample type and pathogen reporting thresholds. Overall for specimens negative by BioFire PN*plus* Panel but positive by SOC culture for which either a semiquantitative or quantitative result was provided, 45.1% had quantities reported by SOC (e.g., 1+, rare, 1 colony, < 10^3^) that would be considered below the level of detection of the BioFire PN*plus* Panel.

*C. pneumoniae*, *M. pneumoniae*, and *L. pneumophila* were detected in 0.28%, 1.66%, and 2.23% of all specimens, respectively. Sensitivities of *C. pneumoniae*, *M. pneumoniae*, and *L. pneumophila* detections by BioFire PN*plus* Panel were 100% (7/7), 90.24% (37/41), and 89.09% (49/55) respectively, and SOC were 57.14% (4/7), 48.78% (20/41), and 89.09% (49/55), respectively (Table 1). Detection of *M. pneumoniae* was significantly better by BioFire PN*plus* Panel than SOC (*p* = 0.0001). BioFire PN*plus* Panel and SOC detection rates for atypical bacteria combined were 90.29% (93/103) and 70.87% (73/103), respectively (*p* = .0008). BioFire PN*plus* Panel detected 97.94% (618/631) of the total viral detections and SOC 21.24% (134/631) (*p* ≤ 0.0001). RV/EV was the predominant virus group detected (58.32%), followed by PIVs (10.78%) and the non-SARS-CoVs (8.24%).

### Single and codetections for BioFire PN*plus* pathogens identified by BioFire PN*plus* and SOC

Distribution of negative and positive results for all specimens and breakdown of composition for BioFire PN*plus* Panel and SOC are shown in Figs. 2. and 1. Majority of BioFire PN*plus* Panel results (70.57%) and SOC results (55.75%) contained at least one bacteria, and at least one virus was detected in 20.49% and 2.93% of specimens, respectively. Mean number of bacteria and viruses detected by BioFire PN*plus* Panel in all specimens, BLS, and SLS were 1.99, 1.8, and 2.15, respectively, compared to 1.44, 1.44, and 1.8 respectively, by SOC (Table 2). For all specimens, 73.81% of BioFire PN*plus* Panel positive specimens (*n* = 1875) contained 1 (45.22%) or 2 (28.59%) pathogens compared to SOC positive specimens (*n* = 1380) where 92.32% contained 1 (65.51%) or 2 (26.81%) BioFire PN*plus* Panel pathogens.

**Table 2:** Number of pathogens (contained in the BioFire PN*plus* Panel) detected by BioFire Pneumonia *plus* (PN*plus*) Panel versus standard of care (SOC)

Sample type	Method	Number positive	1 pathogen, number (%)	2 pathogens, number (%)	3 pathogens, number (%)	4 pathogens, number (%)	5 pathogens, number (%)	6+ pathogens, number (%)	Mean number per sample
All specimens	PN*plus*	1875	848 (45.22)	536 (28.59)	275 (14.67)	130 (6.93)	60 (3.20)	26 (1.39)	1.99
	SOC	1380	904 (65.51)	370 (26.81)	85 (6.16)	15 (1.09)	4 (0.29)	2 (0.14)	1.44
BAL-like specimens	PN*plus*	855	446 (52.16)	239 (27.95)	100 (11.70)	43 (5.03)	22 (2.57)	5 (0.59)	1.8
	SOC	640	432 (67.50)	200 (31.25)	4 (0.78)	3 (0.47)	0 (0.0)	0 (0.0)	1.44
Sputum-like specimens	PN*plus*	1020	402 (39.41)	297 (29.12)	175 (17.16)	87 (8.53)	38 (3.73)	21 (2.06)	2.15
	SOC	740	472 (63.78)	199 (26.89)	56 (7.57)	10 (1.35)	1 (0.14)	2 (0.27)	1.8

*BAL-like* bronchoalveolar lavage-like

Among the 1027 BioFire PN*plus* Panel specimens with codetections, 63.0% (647/1027) had only multiple bacteria (2 to 8); 35.44% (364/1027) had both bacteria (1 to 7) and virus(es) (1 to 3); and 1.33% (16/1207) had only 2 viruses detected. Among the 476 SOC specimens with codetections, 86.34% (411/476) had only multiple bacteria (2 to 5); 13.03% (62/476) had both bacteria (1 to 5) and virus(es) (1 to 2); and 0.63% (3/476) had 3 viruses only detected.

### Pathogens detected by SOC not included in BioFire PN*plus* Panel

Excluding normal oral flora, 649 additional potential pathogens were detected by SOC including 230 gram-negative bacteria, the most prevalent being *Stenotrophomonas maltophilia* (*n* = 70), *Citrobacter* spp. (n = 31), *Acinetobacter* spp. (*n* = 28), *Haemophilus parainfluenzae* (*n* = 22), *Morganella morganii* (*n* = 15), and *Hafnia alvei* (*n* = 11). Another 28 gram-negative bacterial spp., representing 18 genera, were reported less than 10 times. Yeasts were reported for 283 specimens, *Candida* spp. (*n* = 123), *Candida albicans* (*n* = 113), *C. glabrata* (*n* = 27), *C. parapsilosis* (*n* = 6), *C. krusei* (*n* = 5), *C. tropicalis* (*n* = 5), *C. kefyr* (*n* = 2), and *C. lusitaniae* (*n* = 2). *Pneumocystis* sp. was reported for 11 specimens, molds for 28 specimens, including 11 *Aspergillus fumigatus*, and five anaerobes including three *Fusobacterium* spp. There were 42 gram-positive cocci of questionable significance including 24 *Enterococcus* spp. and seven *Streptococcus* C/G groups. There were 24 gram-positive rods, including 15 *Corynebacterium* spp. and 1 *Nocardia* sp. and four *Mycobacterium* (two *M. tuberculosis*, one *M. avium*, and one *M. fortuitum*). Additional viral targets (*n* = 22) included cytomegalovirus (*n* = 10), herpes simplex virus (*n* = 5), Epstein-Barr virus (*n* = 4), human herpes virus 6 (*n* = 2), and varicella zoster virus (*n* = 1).

Including BioFire PN*plus* Panel pathogens and additional SOC pathogens not in BioFire PN*plus* Panel, the number of potential pathogens for all specimens totaled 4542, of which BioFire PN*plus* Panel detected 82.41% (3743/4542) and SOC detected 58.21% (2644/4542) (*p* ≤ 0.0001). Excluding pathogens of questionable significance that may be considered colonizers (yeasts, gram-positive cocci, gram-positive rods [excluding *Mycobacteria* spp., *Nocardia* spp.], molds [due to difficulty in interpretation even in immunocompromised persons]), the total number of potential pathogens was 4274 of which BioFire PN*plus* Panel detected 87.58% (3743/4274) and SOC detected 55.59% (2376/4274) (*p* ≤ 0.0001). The NPVs for individual pathogens not included in the BioFire PN*plus* Panel ranged from 97.16% for *S. maltophilia* to 99.96% for organisms reported only once. The composite NPV of the BioFire PN*plus* Panel for all off-panel pathogens minus those of questionable significance was 89.12%. This evaluation did not consider the quantity of the off-panel pathogens reported and the NPV may in practice be higher based on laboratory sample type and pathogen reporting thresholds.

### Concordance of BioFire PN*plus* Panel and SOC results

Overall, 1209/2463 specimens (49.10%) demonstrated total concordance between BioFire PN*plus* Panel and SOC results (Table 3). This included positive concordance (27.0%), negative concordance (5.36%) for BioFire PN*plus* Panel bacteria and viruses, and 16.73% for SOC specimens positive for non-BioFire PN*plus* Panel bacteria and viruses. 26.43% (651/2463) of specimens demonstrated partial concordance and 24.48% (603/2463) no concordance. Positive, partial, and negative concordances for BLS were 56.85%, 20.55%, and 22.59%, respectively, and 41.13%, 32.34%, and 26.35%, respectively, for SLS.

**Table 3:** Concordance of BioFire PN*plus* (PN*plus*) Panel and standard of care (SOC) results for all specimens, BAL-like specimens, and sputum-like specimens

	All specimens		BAL-like specimens		Sputum-like specimens	
	Total number (2463)	Percentage of total	Total number (1226)	Percentage of total	Total number (1237)	Percentage of total
Overall concordance^a^	1209	49.10	697	56.85	511	41.31
Overall concordant positive^b^	665	27.00	353	28.79	311	25.15
Concordant bacteria^c^	601	24.40	320	26.10	280	22.64
Concordant bacteria and virus^d^	25	1.02	6	0.49	19	1.54
Concordant virus^e^	39	1.58	27	2.20	12	0.97
Concordant negative for any pathogen^f^	412	16.73	257	20.96	155	12.53
Concordant negative for PN*plus* pathogens^g^	132	5.36	87	7.10	45	3.64
Partial concordance^h^	651	26.43	252	20.55	400	32.34
No concordance^i^	603	24.48	277	22.59	326	26.35

^a^Overall concordance: samples fully concordant for analytes detected by PN*plus* Panel and SOC and for samples reported as negative by both methods

^b^Concordant positive: PN*plus* Panel and SOC in agreement for all pathogens detected

^c^Concordant bacteria: PN*plus* Panel and SOC in agreement for all bacteria detected

^d^Concordant bacteria and virus: PN*plus* Panel and SOC in agreement for all bacteria and viruses

^e^Concordant virus: PN*plus* Panel and SOC in agreement for all viruses detected

^f^Concordant negative for any pathogen: PN*plus* Panel and SOC both reported as negative for any pathogen

^g^Concordant negative for PN*plus* Panel analytes: PN*plus* Panel and SOC negative for pathogens on PN*plus* Panel (other pathogens may have been detected by SOC)

^h^Partial concordance: PN*plus* Panel and SOC in agreement for some pathogens detected

^i^No concordance: PN*plus* Panel and SOC not in agreement for pathogens in PN*plus* Panel

*BAL-like specimens* bronchoalveolar lavage-like specimens

### Bacterial quantification by BioFire PN*plus* Panel and SOC

Semiquantitative results for BioFire PN*plus* Panel and SOC bacteria were compared for 1297 matched detections (Table 4). Mean differences between all three levels of SOC SRVs and BioFire PN*plus* Panel SRVs for all bacteria, BLS bacteria, and SLS bacteria were 1.06 (range 0.96–1.14), 1.34 (range 1.19 to 1.52), and 0.90 (range 0.71 to 1.14), respectively. For a subset of specimens (*n* = 903) with SOC quantitative culture results, mean difference between SOC SRV and BioFire PN*plus* Panel SRV for bacteria was 1.18. For specimens BioFire PN*plus* Panel positive and SOC negative, mean BioFire PN*plus* Panel SRVs for all bacteria, BLS bacteria, and SLS bacteria were 3.21, 3.10, and 3.27, respectively. Using this subset of BioFire PN*plus* Panel positive/SOC negative results, if a threshold for BLS reporting was set at BioFire PN*plus* Panel bin values of 10^4^, 10^5^, 10^6^, or ≥ 10^7^, then 482, 290, 158, and 77 BioFire PN*plus* Panel bacteria results, respectively, would be reported as positive and discordant with SOC. If a threshold for SLS reporting was set at BioFire PN*plus* Panel bin values of 10^4^, 10^5^, 10^6^, or ≥ 10^7^, then 834, 540, 336, and 177 bacteria results, respectively, would be reported as discordant with SOC. Therefore, increasing the BioFire PN*plus* Panel bin threshold for reporting bacteria as detected would result in increased concordance with SOC reported as culture negative. Establishment of reporting guidelines in consideration of both BioFire PN*plus* Panel bin values and the inherent log value differences between BioFire PN*plus* Panel results and SOC results improve overall concordance of bacteria reporting.

**Table 4:** Comparison of standard of care (SOC) bacteria standard reference values (SRV) and BioFire Pneumonia *plus* (PN*plus*) Panel bacteria standard reference values

		SOC level 1	SOC level 2	SOC level 3	PN*plus* value	Mean delta, PN*plus*/SOC^a^	PN*plus* value, SOC ND^b^
All detections (*N* = 1297)	Mean SRV^c^	2.94	3.19	3.32	4.27		3.21
	Log delta^d^	1.14	1.09	0.96		1.06	
BAL-like detections (*N* = 645)	Mean SRV	2.64	2.85	2.97	4.16		3.10
	Log delta	1.52	1.31	1.19		1.34	
Sputum-like detections (*N* = 652)	Mean SRV	3.25	3.53	3.68	4.39		3.27
	Log delta	1.14	0.86	0.71		0.90	

The standardized reference values (SRV) (1–5) were derived from three different interpretation levels (defined in Supplemental Table 6)

^a^Combined mean difference between the BioFire PN*plus* SRVs and SOC SRVs for all 3 interpretation levels

^b^Mean SRV for bacteria detected by BioFire PN*plus* Panel but not detected by SOC

^c^Mean SRV for all samples per interpretation level 1, 2, or 3

^d^Difference between BioFire PN*plus* Panel SRV and SOC SRV (descriptive, numerical, and quantitative) per interpretation level

*BAL-like* bronchoalveolar lavage-like

SOC SRVs and BioFire PN*plus* Panel SRVs were equivalent for 25.37% of all bacteria, 19.38% of BLS bacteria, and 31.92% of SLS bacteria (Table 5). BioFire PN*plus* Panel SRVs were greater than SOC SVRs for 69.55% of all bacteria, 76.28% of BLS bacteria, and for 62.88% of SLS bacteria, with majority of BioFire PN*plus* Panel SRVs 1–2 logs greater than SOC SVRs. SOC SVRs were greater than BioFire PN*plus* Panel SRVs for 5.09% of all bacteria, 4.34% of BLS bacteria, and 5.83% of SLS bacteria, with majority demonstrating a one log difference.

**Table 5:** Distribution of standard of care (SOC) bacteria standardized reference values (SRV) and BioFire Pneumonia *plus* (PN*plus*) Panel bacteria standard reference values

	PN*plus* SRV = SOC SRV number (%)	PN*plus* SRV 1 log > SOC SRV number (%)	PN*plus* SRV 2 log > SOC SRV number (%)	PN*plus* SRV 3 log > SOC SRV number (%)	PN*plus* SRV 4 log > SOC SRV number (%)	SOC SRV 1 log > PN*plus* SRV number (%)	SOC SRV 2 log > PN*plus* SRV number (%)	SOC SRV 3 log > PN*plus* SRV number (%)
All detections (*N* = 1297)	329 (25.37)	477 (36.78)	282 (21.74)	123 (9.48)	20 (1.54%)	53 (4.09)	8 (0.62)	5 (0.39)
BAL-like detections (*N* = 645)	125 (19.38)	213 (33.02)	189 (29.30)	74 (11.47)	16 (2.48)	24 (3.72)	1 (0.16)	3 (0.49)
Sputum-like detections (*N* = 652)	204 (31.29)	264 (40.49)	93 (14.26)	49 (7.52)	4 (0.61)	29 (4.45)	7 (1.07)	2 (0.31)
Total %	PN*plus* = SOC (25.37)	PN*plus* > SOC (62.88)				SOC > PN*plus* (5.83)		

*BAL-like* bronchoalveolar lavage-like

### Detection of MSSA and MRSA

A total of 531 specimens contained *S. aureus*, 97.93% (520/531) detected by BioFire PN*plus* Panel and 58.95% (313/531) detected by SOC (*p* ≤ 0.0001) with a concordance of 56.87% (Table 6). BioFire PN*plus* Panel did not detect 11 *S. aureus* (2.07%, all MSSA). A total of 24 specimens were reported to contain MSSA by SOC but were reported to contain MRSA by BioFire PN*plus* Panel and two specimens reported to contain MRSA by SOC were reported as MSSA by BioFire PN*plus* Panel. Mean SRVs for *S. aureus* detected by SOC, BioFire PN*plus* Panel, and detected only by BioFire PN*plus* Panel were 3.14, 4.18, and 2.92, respectively.

**Table 6:** Detection of methicillin sensitive *Staphylococcus aureus* (MSSA) and methicillin resistant *S. aureus* (MRSA) by BioFire Pneumonia *plus* (PN*plus*) Panel and standard of care (SOC)

	Total (+) number (%)	PN*plus* (+) number (%)	SOC (+) number (%)	SOC (+) PN*plus* (+) number (%)	SOC (−) PN*plus* (+) number (%)	SOC (+) PN*plus* (−) number (%)
*Staphylococcus aureus* (total)	531 (100)	520 (97.93)	313 (58.95)	302 (56.87)	218 (41.05)	11 (2.07)
*Staphylococcus aureus* (MSSA)		412^a^	269^b^	234	178	11
*Staphylococcus aureus* (MRSA)		106^b^	47^a^	45	59	0
Mean standard SVR^c^	3.14	4.18	2.92			

(+), *S. aureus* detected; (−), *S. aureus* not detected

^a^Two samples reported to contain MRSA by SOC were reported to contain MSSA by BioFire PN*plus* Panel

^b^Twenty-four samples reported to contain just MSSA by SOC were reported to contain MRSA by BioFire PN*plus* Panel

^c^SVR: standard reference value based on interpretation level 2 (refer to Supplemental Table 6)

### Detection of gram-negative resistance markers

BioFire PN*plus* Panel detected in 1537 specimen bacteria (*Acinetobacter baumannii-calcoaceticus* complex, *Enterobacter cloacae* complex, *Escherichia coli*, *Klebsiella aerogenes*, *Klebsiella oxytoca*, *Klebsiella pneumoniae* group, *Proteus* spp., *P. aeruginosa*, *Serratia marcescens*) for which ESBL and/or carbapenemase resistance genes would be reported. Of these, 185 (12.04%) had a total of 229 resistance genes (*bla*_CTX-M_ [*n* = 133)], *bla*_KPC_ [*n* = 67], *bla*_IMP_ [*n* = 1], *bla*_NDM_ [*n* = 2], *bla*_VIM_ [*n* = 26]). Six *bla*_OXA-48-like_ genes were detected in 1029 specimens (0.58%) positive by BioFire PN*plus* Panel for one or more of the following: *E. cloacae* complex, *E. coli*, *K. aerogenes*, *K. oxytoca*, *K. pneumoniae* group, *Proteus* spp., or *S. marcescens*. One resistance gene was detected in 76.76% (142/185) of specimens, two in 20.0% (37/185), and three in 3.24% (6/185). Due to large variations and inconsistencies in reporting of SOC phenotypic/genetic susceptibility data, no comparisons could be made.

## Discussion

This multinational study is the largest to date that compared BioFire PN*plus* Panel to SOC testing, which varied extensively by site and physician prescribing practices. BioFire PN*plus* Panel identified significantly more positive specimens (76.13%) than SOC (56.03%) (*p* ≤ 0.0001) and more potential pathogens than SOC (*p* ≤ 0.0001) independent of specimen type. Largest discrepancies for bacterial detections were for fastidious pathogens, which may be concealed by overgrowth of normal flora, or may be non-viable, including *Streptococcus pyogenes* (77.27%), *Streptococcus agalactiae* (78.85%), *Moraxella catarrhalis* (61.7%), *H. influenzae* (53.97%), and *S. pneumoniae* (48.07%). Despite detection differences, the three most common bacteria identified by BioFire PN*plus* Panel and SOC were similar, *S. aureus*, *H. influenzae*, and *P. aeruginosa*. Lower SOC bacteria detection may relate to local reporting guidelines and testing of specimens from patients on antimicrobials. The most prevalent pathogen not included on BioFire PN*plus* Panel detected by SOC was *S. maltophilia*; however, the incidence was low (2.84%), similarly with other various gram-negative rods (6.5%). Despite the low prevalence of pathogens not detected by BioFire PN*plus* Panel, it is essential to perform culture or other ancillary testing to detect off-panel pathogens and to provide susceptibility results.

Our rate of bacteria detection (95.77%) as compared to culture was consistent with Mitton et al. (92.0%) [21] and Yoo et al. (99.3%) [22]. A study by Webber et al. demonstrated that the BioFire PN*plus* Panel identified most bacteria (98.4%) detected by SOC and additionally 92 bacteria including more *S. aureus* (23.9%) and *H. influenza* (27.2%) [23]. Murphy et al. identified potential pathogens in 48.82% of BLS and in 72.01% of SLS with BioFire PN Panel, detecting more *S. aureus*, *H. influenzae*, *M. catarrhalis*, and *P. aeruginosa* [20]. In this study, the overall BioFire PN*plus* Panel PPA and NPA for common bacteria were 92.90% and 96.10%, respectively, and similar to other studies. Clinical studies for FDA clearance demonstrated a PPA of 96.2% and NPA of 98.3% for BLS and a PPA of 96.3% and NPA of 97.2% for SLS after discordant resolution using a molecular comparator. Lee et al. demonstrated a PPA of 90% and NPA of 97.4% as compared to SOC bacteria detection, with BioFire PN Panel identifying a pathogen in 47.4% of BLS and in 60% of SLS for an overall positivity rate of 55.93% [24]. Edin demonstrated for on-panel pathogens a PPA of 100% and an NPA of 73.2% [25], and Gastli demonstrated a PPA of 94.4% and an NPA of 96.0% as compared to culture [26]. Similarly, a VAP study using a research use only version of BioFire PN*plus* Panel demonstrated for bacteria an 89.0% PPA and 95.9% NPA with SOC [27]. BioFire PN*plus* Panel reflects high performance compared to SOC, yielding additional clinically actionable results that may be missed by SOC.

Prevalence of atypical pathogens in this specimen set was low (4.17%), with *L. pneumophila* the most frequently detected. Low SOC percent detections of *M. pneumoniae* (48.78%) and *C. pneumoniae* (57.14%) compared to BioFire PN*plus* Panel (90.24% and 100%, respectively) were mainly due to lack of testing and therefore a missed opportunity in CAP to either limit treatment to a macrolide or fluoroquinolone or stop treatment if tested negative. This missed opportunity for applying antimicrobial stewardship principles and streamlining therapy is of particular importance due to adverse effects of fluoroquinolones [28]. Of the 41 patients *M. pneumoniae* positive, age was available for 31 (range 7 to 88 years), including 12 (range 23 to 80 years) with no SOC result. Lack of testing may be a study artifact or be indicative of local testing practices. Adults may not be tested as *M. pneumoniae* is often viewed as an illness of school age children, adolescents, and young adults, although studies demonstrate infections in all aged adults, with up to a 15% prevalence in persons aged 56 or older [29]. Conversely, percentage of *L. pneumophila* detected by BioFire PN*plus* Panel and SOC were similar (89.09%). There were only 6/55 specimens *L. pneumophila* positive with no SOC result, indicating a higher awareness compared to *M. pneumoniae* and *C. pneumoniae*.

BioFire PN*plus* Panel detections were limited for certain viruses (example, influenza A, influenza B) due to seasonality and time of specimen collection. SOC viral detections were additionally limited by lack of testing. Webber et al. compared standard viral testing with BioFire PN*plus* Panel and demonstrated a 99.2% correlation [23]. Hughes et al. found an 87% PPA and 100% NPA for BioFire PN*plus* Panel viral detections compared to BioFire® FilmArray® Respiratory 2 (RP2) Panel (BioFire Diagnostics, LLC) [30]. Virus detection in absence of a bacterial pathogen and in conjunction with clinical presentation, chest radiograph, and other diagnostic tests, such as a low procalcitonin, could support antimicrobial stewardship and discontinuation of antibiotics in the setting of CAP [31].

BioFire PN*plus* Panel identified more codetections (41.85%) compared to SOC (19.21%), which was mainly influenced by lack of SOC viral testing. Codetections were commonly identified by Webber et al. (25%) [23], Murphy et al. (29.49%) [20], and Lee et al. (42.3%) [24]. All three studies found the majority of codetections contained 2 pathogens, but could rarely contain 5 to 6+ pathogens, similar to our results. In this study, BioFire PN*plus* Panel on average identified more potential pathogens (1.99) per specimen than SOC (1.44). Detection of multiple pathogens raises interpretation questions that need to be viewed in light of clinical parameters, pathogens detected, and abundances.

Use of BioFire PN*plus* Panel has led to concerns that identification of more bacteria than SOC, which may be colonizers, could lead to antibiotic overtreatment. Specimen types should be considered. SLS are prone to more oropharyngeal contamination compared to BLS. *H. influenzae*, *S. pneumoniae*, and *M. catarrhalis* can be normal flora, and hospitalized or ventilated patients may be colonized with gram-negative bacilli and *S. aureus*. Although laboratory reporting varies depending on specimen type, patient populations, and clinical need, clinical guidelines recommend using different reporting thresholds for different specimen types. Generally, SLS bacteria are considered, in light of other variables, significant at ≥ 10^6^ or ≥ 10^7^ (moderate, numerous, 3+, 4+) and in BLS ≥ 10^3^ or ≥ 10^4^ (few, moderate, 2+ or greater). For this reason, the BioFire PN*plus* Panel lower limit of reporting for bacteria was set at 10^3.5^ genomes/mL. The bin values may allow for different interpretations based on specimen type. For example, SOC and BioFire PN*plus* Panel concordance for bacteria in SLS was 51.23% but when only evaluating BioFire PN*plus* Panel results reported at 10^6^ and ≥ 10^7^, correlation increased to 66.8%. Additionally, more BioFire PN*plus* Panel negative/SOC negative results would be obtained when higher bin thresholds for reporting BioFire PN*plus* Panel bacteria are used. Overall, the mean SRVs for SOC were consistently ~ 1 log lower than BioFire PN*plus* Panel SRV when using three different interpretation schemes. Mean SRV for specimen culture negative, BioFire PN*plus* Panel positive, was 3.21 and would potentially equate to SOC SRV of 2.21, which likely is below the limit of detection or limit of culture reporting. Similarly, SOC *S. aureus*-negative specimens but BioFire PN*plus* positive had a SRV of 2.92.

Murphy et al. demonstrated that BioFire PN Panel bin values were accurate and reproducible within ± 0.5 log_10_ copies/mL and correlated with another quantitative molecular method [20]. Despite low concordance with quantitative culture, particularly when values were < 10^6^ (3.1–38.9%), concordance improved to 90.9–100% when quantitative culture values were > 10^6^. There were few instances when BioFire PN Panel did not detect a bacterium or reported values lower than quantitative culture, which is similar to what we report in this study. Lee et al. also demonstrated an overestimation of quantification by BioFire PN Panel [24]. Buchan et al. demonstrated that PN values were frequently higher than culture values, resulting in semiquantitative agreement (within the same log_10_ value) of 43.6% [32]. Gastli et al. reported that 90.1% of organisms with a BioFire PN*plus* Panel result of ≥ 10^6^ grew significantly in culture [26] and Yoo et al. reported that 86% of bacteria considered significant by culture (moderate or many quantities) yielded BioFire PN*plus* Panel results of ≥ 10^7^ [22 not 26] 22.

Although BioFire PN*plus* Panel does not make claims as to the significance of the bin value, knowing the relative bacteria abundance may be helpful in understanding coinfections and differentiating colonization versus infection. BioFire PN*plus* Panel bin values should be interpreted in consideration of specimen type, Gram stain, types and bin values for other pathogens detected, type of pneumonia, presence of resistance markers, biomarkers, and clinical risk factors. Laboratories should establish reporting guidelines and provide physician education in conjunction with a multidisciplinary team consisting of ID, critical care specialists, pulmonologists, stewardship committee, and infection control practitioners.

Major limitations of this study were a lack of clinical information and comprehensive gram-negative phenotypic antibiotic susceptibility data, such as that described by Murphy et al. [20], needed to better understand the relevance of resistance marker results and impact on patient care. Discordant analyses were not performed due to the large number of participating sites and the number of specimens tested. Discordant analyses would have required some type of confirmatory testing for 150 culture isolates (BioFire PN*plus* Panel negative) and 1898 individual PCRs or direct sample sequencing for the confirmation of pathogens only detected by BioFire PN*plus* Panel (SOC negative). However, this study therefore does highlight the need for microbiologists and clinicians to address issues relating to discordant results and test interpretation. Additionally, detailed information regarding testing for viral or atypical bacterial pathogens for each sample was not available so no direct performance comparison could be made. Gram stains were not systematically reported and it is not known if a quality score was a testing requirement. However, considering the high sensitivity and specificity of BioFire PN*plus* Panel as demonstrated in the US FDA clinical studies [20], in combination with detection of key gram-positive and gram-negative resistance genes, BioFire PN*plus* Panel may facilitate decisions to optimize therapy, including discontinuation, de-escalation, and escalation, which should be evaluated on a patient-by-patient basis. Depending on local epidemiology, negative results for drug-resistant pathogens may yield a high negative predictive value and may allow for therapy de-escalation in the right clinical context, an approach endorsed by IDSA CAP guidelines and in CDC Core Elements of Hospital Antibiotic Stewardship Programs [6, 33]. Similarly, several hypothetical therapeutic reviews based on BioFire PN Panel results as compared to actual treatment revealed potential for antibiotic adjustment. Buchan et al. found, based on BioFire PN Panel results, 70.7% of patients could have had an antibiotic adjustment, including discontinuation or de-escalation in 48.2% resulting in an average savings of 6.2 antibiotic days/patient [32]. Lee et al. identified a potential for BioFire PN Panel to alter antibiotic prescription in 40.7% of patients [24]. A study by Monard et al. demonstrated that a multidisciplinary committee proposed modifications of empiric therapy in 77% of pneumonia episodes, including de-escalation (40%) and escalation (22%), and in microbiologic documented cases, the BioFire PN*plus* Panel increased appropriateness of therapy in 87% of cases as compared to 77% in routine care [34]. This data provides an early indication that proper use and interpretation of BioFire PN*plus* Panel could lead to targeted therapy not an increase in inappropriate antimicrobial usage. Prospective interventional studies in progress will provide data on interpretation of bin values, detection of resistance genes, and clinical impact of a rapid diagnosis. Finally, the BioFire PN*plus* bin comparison to SOC reporting was difficult to standardize as culture reporting can vary from technologist to technologist and laboratory to laboratory, which could lead to interpretive error. However, despite using 3 different interpretative criteria, results did not significantly differ. Strengths of the study include the large specimen size, the even distribution of specimen types, and geographical diversity of testing sites with differences in both ordering practices and results reporting.

The clinical laboratory plays a vital role in diagnosis of CAP, HAP, and VAP but faces numerous challenges due to testing complexity [1. 5, 8-10]. Often poor-quality sputum specimens are submitted and without quality rejection screening by Gram stain, culture results can be misleading or negative. HAP and VAP patients pose a different dilemma since these patients quickly become colonized with *S. aureus* and various gram-negative bacilli which may lead to pneumonia with multidrug-resistant strains [15, 16]. Specimens can contain a diversity of pathogens including bacteria, viruses, and fungi [4, 6–10, 34–38]. Time to traditional bacterial detection is 24–72 h and antibiotic susceptibility data takes an additional 24–48 h. Comprehensive viral diagnostics are often not performed aside from influenza A/B testing, may not be performed 24/7, or require referral to a reference laboratory delaying time to results. Urinary antigen tests for *S. pneumoniae* provide results in < 30 min but can be false negative and false positive, particularly in children [39]. Urinary antigen tests for *L. pneumophila* are restricted in serotype detection and can have sensitivities of < 50% [40, 41]. Serology can be difficult to interpret and may require an acute and convalescent serum collected weeks apart. NAATs are the gold standard for the detection of the atypical bacteria but may not be routinely performed. Consequently, testing imitations lead to empiric HAP/VAP treatment with broad-spectrum antibiotics, especially in regions with high antimicrobial resistance rates. The switch to targeted therapy can take days, increasing risk of antimicrobial resistance and adverse events such as acute kidney injury and *C. difficile* disease [18]. Finally, if specimens are obtained after the start of antibiotic treatment, results may be altered or negative, without identifying the etiologic agent [5].

In conclusion, the BioFire PN*plus* Panel meets the challenges associated with routine test methods including poor pathogen recovery, lack of diagnostic comprehensiveness, and delayed time to result [42]. However, several factors need to be considered including the lack of a specimen quality marker and the inability to report the presence or absence of normal flora [43]. Although a Gram stain is not required prior to testing, good laboratory practice should still be followed to insure sample quality [43]. Pretreatment or dilution of samples would affect both the sensitivity of the assay and semiquantitative results and therefore is not recommended in the manufacturer’s instructions for use. Interpretation challenges include understanding the increased detection rates, significance of the bin value, the differentiation between colonization and infection, and the presence of gram-negative resistance markers without direct linkage to a specific pathogen. However, approved for use with BLS and SLS, BioFire PN*plus* allows for easy specimen testing for CAP [6, 7] and meets IDSA/ATS recommendations [13] for non-invasive diagnostic testing as a preferred method for VAP and ERS guidelines to test distal quantitative specimens [16]. Additionally, studies that used BioFire PN*plus* panels in COVID-19 patients demonstrated not only improved diagnosis of bacterial coinfections but enhanced options for appropriate therapy [35, 44, 45]. Verroken et al. demonstrated that BioFire PN*plus* Panel speeded up therapeutic changes in 46.9% of COVID-19 patients, five patients having antibiotics stopped and one third remained antibiotic free [35]. BioFire PN*plus* is rapid, simple to perform, and highly robust, with only 0.53% of the specimens in this study yielding invalid results. Detection of pathogens and antibiotic resistance markers can be used to inform immediate treatment decisions and improve patient outcomes.

## Supplementary Information

ESM 1 (DOCX 19 kb)

ESM 2 (DOCX 21 kb)

ESM 3 (DOCX 18 kb)

ESM 4 (DOCX 18 kb)

## Data Availability

The datasets generated during and/or analyzed during the current study are not publicly available due to restrictions by individual contributors but are available as composite data from the corresponding author on reasonable request.
