# Supplementary material for: Multinational evaluation of the BioFire® FilmArray® Pneumonia plus Panel as compared to standard of care testing
Source: Eur J Clin Microbiol Infect Dis. 2021 Mar 2;40(8):1609–22. doi: 10.1007/s10096-021-04195-5 (PMC7924818; doi:10.1007/s10096-021-04195-5)
Supplement: Supplementary file 1 — (DOCX 19 kb) [file 10096_2021_4195_MOESM1_ESM.docx]

Multi-national Evaluation of the BioFire^®^ FilmArray^®^ Pneumonia *plus* Panel as Compared to Standard of Care Testing

European Journal of Clinical Microbiology and Infection

Christine C Ginocchio^1,2*^, Carolina Garcia-M^3^, Barbara Mauerhofer^3^, Cory Rindlisbacher^1^ and the EME Evaluation Program Collaborative

^1^.BioFire Diagnostics, LLC, Salt Lake City, UT, USA. ^2.^ bioMérieux, USA, ^3.^ bioMérieux, Marcy l’Etoile, France

*Corresponding author: Christine C Ginocchio

bioMéríeux/BioFire Diagnostics

515 Colorow Way

Salt Lake City , UT 84108

Phone: +1 919-638-0668

[christine.ginocchio@biomerieux.com](mailto:christine.ginocchio@biomerieux.com)

ORCID: 0000-0002-8200-0324

Supplemental Table 7 Distribution of Study Specimens by Country and Site

| Country | Total Specimens By Country | | | | Total Specimens By Site | | | | |
| --- | --- | --- | --- | --- | --- | --- | --- | --- | --- |
|  | | SLS | BLS | Total | | SLS | BLS | Total |  |
| Austria | | 5 | 7 | 12 | | 5 | 7 | 12 |  |
| Belgium | | 42 | 55 | 97 | |  |  |  |  |
| Belgium-1 | |  |  |  | | 29 | 9 | 38 |  |
| Belgium-2 | |  |  |  | | 13 | 46 | 59 |  |
| Denmark | | 54 | 0 | 54 | | 54 | 0 | 54 |  |
| France | | 257 | 277 | 534 | |  |  |  |  |
| France-1 | |  |  |  | | 18 | 40 | 58 |  |
| France-2 | |  |  |  | | 22 | 7 | 29 |  |
| France-3 | |  |  |  | | 30 | 29 | 59 |  |
| France-4 | |  |  |  | | 13 | 24 | 37 |  |
| France-5 | |  |  |  | | 50 | 10 | 60 |  |
| France-6 | |  |  |  | | 5 | 55 | 60 |  |
| France-7 | |  |  |  | | 0 | 59 | 59 |  |
| France-8 | |  |  |  | | 60 | 0 | 60 |  |
| France-9 | |  |  |  | | 20 | 37 | 57 |  |
| France-10 | |  |  |  | | 16 | 15 | 31 |  |
| France-11 | |  |  |  | | 23 | 1 | 24 |  |
| Germany | | 49 | 64 | 113 | |  |  |  |  |
| Germany-1 | |  |  |  | | 23 | 34 | 57 |  |
| Germany-2 | |  |  |  | | 26 | 30 | 56 |  |
| Israel | | 82 | 17 | 99 | |  |  |  |  |
| Israel-1 | |  |  |  | | 45 | 12 | 57 |  |
| Israel-2 | |  |  |  | | 37 | 5 | 42 |  |
| Italy | | 181 | 289 | 470 | |  |  |  |  |
| Italy-1 | |  |  |  | | 25 | 33 | 58 |  |
| Italy-2 | |  |  |  | | 10 | 48 | 58 |  |
| Italy-3 | |  |  |  | | 43 | 15 | 58 |  |
| Italy-4 | |  |  |  | | 60 | 42 | 102 |  |
| Italy-5 | |  |  |  | | 0 | 37 | 37 |  |
| Italy-6 | |  |  |  | | 8 | 35 | 43 |  |
| Italy-7 | |  |  |  | | 35 | 23 | 58 |  |
| Italy-8 | |  |  |  | | 0 | 56 | 56 |  |
| Netherlands | | 57 | 0 | 57 | | 57 | 0 | 57 |  |
| Portugal | | 6 | 52 | 58 | | 6 | 52 | 58 |  |
| Spain | | 199 | 264 | 463 | |  |  |  |  |
| Spain-1 | |  |  |  | | 18 | 24 | 42 |  |
| Spain-2 | |  |  |  | | 21 | 13 | 34 |  |
| Spain-3 | |  |  |  | | 26 | 23 | 49 |  |
| Spain-4 | |  |  |  | | 25 | 22 | 47 |  |
| Spain-5 | |  |  |  | | 23 | 31 | 54 |  |
| Spain-6 | |  |  |  | | 3 | 39 | 42 |  |
| Spain-7 | |  |  |  | | 9 | 37 | 46 |  |
| Spain-8 | |  |  |  | | 27 | 19 | 46 |  |
| Spain-9 | |  |  |  | | 0 | 36 | 36 |  |
| Spain-10 | |  |  |  | | 34 | 12 | 46 |  |
| Spain-11 | |  |  |  | | 11 | 1 | 12 |  |
| Spain-12 | |  |  |  | | 2 | 7 | 9 |  |
| Sweden | | 131 | 20 | 151 | |  |  |  |  |
| Sweden-1 | |  |  |  | | 68 | 18 | 86 |  |
| Sweden-2 | |  |  |  | | 63 | 2 | 65 |  |
| Switzerland | | 87 | 111 | 198 | |  |  |  |  |
| Switzerland-1 | |  |  |  | | 28 | 26 | 54 |  |
| Switzerland-2 | |  |  |  | | 41 | 28 | 69 |  |
| Switzerland-3 | |  |  |  | | 0 | 45 | 45 |  |
| Switzerland-4 | |  |  |  | | 18 | 12 | 30 |  |
| United Kingdom | | 87 | 70 | 157 | |  |  |  |  |
| United Kingdom-1 | |  |  |  | | 31 | 0 | 31 |  |
| United Kingdom-2 | |  |  |  | | 0 | 27 | 27 |  |
| United Kingdom-3 | |  |  |  | | 10 | 20 | 30 |  |
| United Kingdom-4 | |  |  |  | | 0 | 18 | 18 |  |
| United Kingdom-5 | |  |  |  | | 46 | 5 | 51 |  |
| Total | | 1237 | 1226 | 2463 | | 1237 | 1226 | 2463 |  |

Abbreviations: SLS, sputum-like specimens; BLS, bronchoalveolar lavage-like specimens
