# Supplementary material for: Multinational evaluation of the BioFire® FilmArray® Pneumonia plus Panel as compared to standard of care testing
Source: Eur J Clin Microbiol Infect Dis. 2021 Mar 2;40(8):1609–22. doi: 10.1007/s10096-021-04195-5 (PMC7924818; doi:10.1007/s10096-021-04195-5)
Supplement: Supplementary file 2 — (DOCX 21 kb) [file 10096_2021_4195_MOESM2_ESM.docx]

Multi-national Evaluation of the BioFire^®^ FilmArray^®^ Pneumonia *plus* Panel as Compared to Standard of Care Testing

European Journal of Clinical Microbiology and Infection

Christine C Ginocchio^1,2*^, Carolina Garcia-M^3^, Barbara Mauerhofer^3^, Cory Rindlisbacher^1^ and the EME Evaluation Program Collaborative

^1^.BioFire Diagnostics, LLC, Salt Lake City, UT, USA. ^2.^ bioMérieux, USA, ^3.^ bioMérieux, Marcy l’Etoile, France

*Corresponding author: Christine C Ginocchio

bioMéríeux/BioFire Diagnostics

515 Colorow Way

Salt Lake City , UT 84108

Phone: +1 919-638-0668

[christine.ginocchio@biomerieux.com](mailto:christine.ginocchio@biomerieux.com)

ORCID: 0000-0002-8200-0324

Supplemental Table 8 Standardized reference value (SVR) scoring groups for standard of care (SOC) and BioFire Pneumonia *plus* (PN*plus*) Panel reporting

| Scoring  Scheme  Group | Standardized Reference Value | SOC Semi-Quantitative  Numerical Value | SOC Semi-  Quantitative  Descriptive Value | SOC Quantitative Culture | PN*plus* Semi-Quantitative Bin Value |
| --- | --- | --- | --- | --- | --- |
| 1 | 1 | 1+ | rare | <10^4 |  |
|  | 2 | 2+ | few, occasional | >10^4-<10^5 | 10^4 |
|  | 3 | 3+ | moderate | >10^5-<10^6 | 10^5 |
|  | 4 | 4+ | many, numerous | >10^6 - <10^7 | 10^6 |
|  | 5 |  | plenty | >10^7 | >10^7 |
|  |  |  |  |  |  |
| 2 | 1 |  | rare | <10^4 |  |
|  | 2 | 1+ | few, occasional | >10^4-<10^5 | 10^4 |
|  | 3 | 2+ | moderate | >10^5-<10^6 | 10^5 |
|  | 4 | 3+ | many | >10^6 - <10^7 | 10^6 |
|  | 5 | 4+ | numerous, plenty | >10^7 | >10^7 |
|  |  |  |  |  |  |
| 3 | 1 |  |  | <10^4 |  |
|  | 2 | 1+ | rare | >10^4-<10^5 | 10^4 |
|  | 3 | 2+ | few, occasional | >10^5-<10^6 | 10^5 |
|  | 4 | 3+ | moderate | >10^6 - <10^7 | 10^6 |
|  | 5 | 4+ | many, numerous, plenty | >10^7 | >10^7 |
|  |  |  |  |  |  |
