# Supplementary material for: Multinational evaluation of the BioFire® FilmArray® Pneumonia plus Panel as compared to standard of care testing
Source: Eur J Clin Microbiol Infect Dis. 2021 Mar 2;40(8):1609–22. doi: 10.1007/s10096-021-04195-5 (PMC7924818; doi:10.1007/s10096-021-04195-5)
Supplement: Supplementary file 3 — (DOCX 18 kb) [file 10096_2021_4195_MOESM3_ESM.docx]

Multi-national Evaluation of the BioFire^®^ FilmArray^®^ Pneumonia *plus* Panel as Compared to Standard of Care Testing

European Journal of Clinical Microbiology and Infection

Christine C Ginocchio^1,2*^, Carolina Garcia-M^3^, Barbara Mauerhofer^3^, Cory Rindlisbacher^1^ and the EME Evaluation Program Collaborative

^1^.BioFire Diagnostics, LLC, Salt Lake City, UT, USA. ^2.^ bioMérieux, USA, ^3.^ bioMérieux, Marcy l’Etoile, France

*Corresponding author: Christine C Ginocchio

bioMéríeux/BioFire Diagnostics

515 Colorow Way

Salt Lake City , UT 84108

Phone: +1 919-638-0668

[christine.ginocchio@biomerieux.com](mailto:christine.ginocchio@biomerieux.com)

ORCID: 0000-0002-8200-0324

Supplemental Table 9 Summary of total, BioFire Pneumonia *plus* (PN*plus*) Panel and standard of care (SOC) detections for bacteria^a^ in bronchoalveolar lavage-like samples (BLS)

|  | Number SOC (+)  PN*plus* (+) | | Number SOC (-) PN*plus* (+) | | Number SOC (+)  PNplus (-) | | Number Total (+) | | Number FA (+) | | Number SOC (+) | | Percentage (%) Detected PN*plus* | | Percentage (%) Detected SOC | | *P value^b^* | |
| --- | --- | --- | --- | --- | --- | --- | --- | --- | --- | --- | --- | --- | --- | --- | --- | --- | --- | --- |
| *Acinetobacter calcoaceticus-baumannii* complex | 37 | 11 | | 1 | | 49 | | 48 | | 38 | | 97.96 | | 77.55 | | 0.0055* | |  |
| *Chlamydia pneumoniae* | 1 | 3 | | 0 | | 4 | | 4 | | 1 | | 100.00 | | 25.00 | | 0.1441 | |  |
| *Enterobacter cloacae* complex | 45 | 30 | | 4 | | 79 | | 75 | | 49 | | 94.94 | | 62.03 | | <0.0001* | |  |
| *Escherichia coli* | 79 | 46 | | 11 | | 136 | | 125 | | 90 | | 91.91 | | 66.18 | | <0.0001* | |  |
| *Haemophilus influenzae* | 74 | 112 | | 11 | | 197 | | 186 | | 85 | | 94.42 | | 43.15 | | <0.0001* | |  |
| *Klebsiella aerogenes* | 11 | 13 | | 5 | | 29 | | 24 | | 16 | | 82.76 | | 55.17 | | 0.0469* | |  |
| *Klebsiella oxytoca* | 9 | 14 | | 8 | | 31 | | 23 | | 17 | | 74.19 | | 54.84 | | 0.1845 | |  |
| *Klebsiella pneumoniae group* | 81 | 29 | | 7 | | 117 | | 110 | | 88 | | 94.02 | | 75.21 | | <0.0001* | |  |
| *Legionella pneumophila* | 21 | 3 | | 2 | | 26 | | 24 | | 23 | | 92.31 | | 88.46 | | 1.00 | |  |
| *Moraxella catarrhalis* | 21 | 29 | | 0 | | 50 | | 50 | | 21 | | 100.00 | | 42.00 | | <0.0001* | |  |
| *Mycoplasma pneumoniae* | 2 | 9 | | 1 | | 12 | | 11 | | 3 | | 91.67 | | 25.00 | | 0.0038* | |  |
| *Proteus* spp. | 13 | 10 | | 0 | | 23 | | 23 | | 13 | | 100.00 | | 56.52 | | 0.0013* | |  |
| *Pseudomonas aeruginosa* | 150 | 31 | | 8 | | 189 | | 181 | | 158 | | 95.77 | | 83.60 | | 0.0002* | |  |
| *Serratia marcescens* | 25 | 17 | | 3 | | 45 | | 42 | | 28 | | 93.33 | | 62.22 | | 0.0010* | |  |
| *Staphylococcus aureus* | 140 | 91 | | 5 | | 236 | | 231 | | 145 | | 97.88 | | 61.44 | | <0.0001* | |  |
| *Streptococcus agalactiae* | 1 | 15 | | 1 | | 17 | | 16 | | 2 | | 94.12 | | 11.76 | | <0.0001* | |  |
| *Streptococcus pneumoniae* | 59 | 47 | | 3 | | 109 | | 106 | | 62 | | 97.25 | | 56.88 | | <0.0001* | |  |
| *Streptococcus pyogenes* | 2 | 7 | | 0 | | 9 | | 9 | | 2 | | 100.00 | | 22.22 | | 0.0037* | |  |
| Total | 771 | 517 | | 70 | | 1358 | | 1288 | | 841 | | 94.85 | | 61.93 | | <0.0001* | |  |

Legend: Abbreviations: (+): positive; (-): negative

^a.^ Bacteria present in the BioFire PN*plus* Panel

^b.^ Significant *P value** = <0.05
