# Supplementary material for: Multinational evaluation of the BioFire® FilmArray® Pneumonia plus Panel as compared to standard of care testing
Source: Eur J Clin Microbiol Infect Dis. 2021 Mar 2;40(8):1609–22. doi: 10.1007/s10096-021-04195-5 (PMC7924818; doi:10.1007/s10096-021-04195-5)
Supplement: Supplementary file 4 — (DOCX 18 kb) [file 10096_2021_4195_MOESM4_ESM.docx]

Multi-national Evaluation of the BioFire^®^ FilmArray^®^ Pneumonia *plus* Panel as Compared to Standard of Care Testing

European Journal of Clinical Microbiology and Infection

Christine C Ginocchio^1,2*^, Carolina Garcia-M^3^, Barbara Mauerhofer^3^, Cory Rindlisbacher^1^ and the EME Evaluation Program Collaborative

^1^.BioFire Diagnostics, LLC, Salt Lake City, UT, USA. ^2.^ bioMérieux, USA, ^3.^ bioMérieux, Marcy l’Etoile, France

*Corresponding author: Christine C Ginocchio

bioMéríeux/BioFire Diagnostics

515 Colorow Way

Salt Lake City , UT 84108

Phone: +1 919-638-0668

[christine.ginocchio@biomerieux.com](mailto:christine.ginocchio@biomerieux.com)

ORCID: 0000-0002-8200-0324

Supplemental Table 10 Summary of total, BioFire Pneumonia *plus* (PN*plus*) Panel and standard of care (SOC) detections for bacteria^a^ in sputum-like samples (SLS)

|  | Number SOC (+)  PN*plus* (+) | | | Number SOC (-) PN*plus* (+) | | Number SOC (+)  PN*plus* (-) | | Number Total (+) | | Number FA (+) | | Number SOC (+) | | Percentage (%) Detected PN*plus* | | Percentage (%) Detected SOC | | *P value^b^* |  |  |  |
| --- | --- | --- | --- | --- | --- | --- | --- | --- | --- | --- | --- | --- | --- | --- | --- | --- | --- | --- | --- | --- | --- |
| *Acinetobacter calcoaceticus-baumannii*  complex | | | 41 | 34 | | 1 | | 76 | | 75 | | 42 | | 98.68 | | 55.26 | | <0.00018 | | |  |
| *Chlamydia pneumoniae* | | | 3 | 0 | | 0 | | 3 | | 3 | | 3 | | 100.00 | | 100.00 | | 1.0000 | | |  |
| *Enterobacter cloacae* complex | | | 57 | 69 | | 6 | | 132 | | 126 | | 63 | | 95.45 | | 47.73 | | <0.0001* | | |  |
| *Escherichia coli* | | | 71 | 67 | | 8 | | 146 | | 138 | | 79 | | 94.52 | | 54.11 | | <0.0001* | | |  |
| *Haemophilus influenzae* | | | 120 | 179 | | 8 | | 307 | | 299 | | 128 | | 97.39 | | 41.69 | | <0.0001* | | |  |
| *Klebsiella aerogenes* | | | 18 | 20 | | 3 | | 41 | | 38 | | 21 | | 92.68 | | 51.22 | | 0.0001* | | |  |
| *Klebsiella oxytoca* | | | 17 | 31 | | 4 | | 52 | | 48 | | 21 | | 92.31 | | 40.38 | | <0.0001* | | |  |
| *Klebsiella pneumoniae* group | | | 98 | 47 | | 8 | | 153 | | 145 | | 106 | | 94.77 | | 69.28 | | <0.0001* | | |  |
| *Legionella pneumophila* | | | 22 | 3 | | 4 | | 29 | | 25 | | 26 | | 86.21 | | 89.66 | | 1.0000 | | |  |
| *Moraxella catarrhalis* | | | 27 | 61 | | 3 | | 91 | | 88 | | 30 | | 96.70 | | 32.97 | | <0.0001* | | |  |
| *Mycoplasma pneumoniae* | | | 14 | 12 | | 3 | | 29 | | 26 | | 17 | | 89.66 | | 58.62 | | 0.0164* | | |  |
| *Proteus* spp. | | | 30 | 25 | | 3 | | 58 | | 55 | | 33 | | 94.83 | | 56.90 | | <0.0001* | | |  |
| *Pseudomonas aeruginosa* | | | 155 | 49 | | 3 | | 207 | | 204 | | 158 | | 98.55 | | 76.33 | | <0.0001* | | |  |
| *Serratia marcescens* | | | 33 | 25 | | 3 | | 61 | | 58 | | 36 | | 95.08 | | 59.02 | | <0.0001* | | |  |
| *Staphylococcus aureus* | | | 162 | 127 | | 6 | | 295 | | 289 | | 168 | | 97.97 | | 56.95 | | <0.0001* | | |  |
| *Streptococcus agalactiae* | | | 6 | 28 | | 1 | | 35 | | 34 | | 7 | | 97.14 | | 20.00 | | <0.0001* | | |  |
| *Streptococcus pneumoniae* | | | 75 | 97 | | 4 | | 176 | | 172 | | 79 | | 97.73 | | 44.89 | | <0.0001* | | |  |
| *Streptococcus pyogenes* | | | 3 | 10 | | 0 | | 13 | | 13 | | 3 | | 100.00 | | 23.08 | | 0.0003* | | |  |
| Total | | | 952 | 884 | | 68 | | 1904 | | 1836 | | 1020 | | 96.43 | | 53.57 | | <0.0001* | | |  |

Legend: Abbreviations: (+): positive; (-): negative

^a.^ Bacteria present in the BioFire PN*plus* Panel

^b.^ Significant *P value** = <0.05
